# Supplementary material for: Dynamic evolution of urban infrastructure resilience and its spatial spillover effects: An empirical study from China
Source: PLoS One. 2023 Mar 16;18(3):e0282194. doi: 10.1371/journal.pone.0282194 (PMC10019656; doi:10.1371/journal.pone.0282194)
Supplement: Attachment — Submitted filename: Response to Reviewers.docx [file pone.0282194.s004.docx]

**Reply to-comments**

Manuscript Number: PONE-D-22-30335

Summary of the major changes and responses to the Reviewers’ comments:

Dear Editor,

On behalf of my co-authors, we thank you very much for giving us an opportunity to revise our manuscript again. We greatly appreciate the editors for their positive constructive comments and suggestions on our manuscript entitled “Dynamic evolution of urban infrastructure resilience and its spatial spillover effects: An empirical study from China”. (ID: PONE-D-22-30335).

We have studied the editors’ comments carefully and addressed them one by one. Attached please find the response to the editor and revised manuscript, which we would like to submit for your kind consideration. All modifications in the manuscript are indicated in red font.

We would like to express our great appreciation to you for your comments on our paper. We look forward to hearing from you.

Thank you and best regards.

Yours sincerely,

Hao Wang

-------------------------

School of Geographic Sciences, Hebei Normal University, Shijiazhuang 050024, China

Tel: 86-031180787632; E-mail: liangyanqing@126.com

**Responses to Anonymous** **Reviewer #1’s Comments:**

**Specific comments:**

**Thanks for your replies - please note the numbers below indicate the order of authors' responses to my previous questions. Q1-OK Q2-the authors highlighted the** **innovation, it should be further improved. Q3-OK Q4-OK Q5-OK.**

***Response:*** On behalf of my coauthors, we greatly appreciate your positive and constructive comments and suggestions on our manuscript. We apologize for insufficiently improving the innovations of the article. In accordance with your suggestions, we have further emphasized the points of innovation of the paper, which mainly focus on three aspects: the indicator system, research perspective, and research scale. The specific information is as follows (page 3, lines 109-116):

“In summary, first, this paper constructs a comprehensive UIR evaluation system by integrating energy, supply and drainage, transport, postal and communication, environmental, and cultural and health facilities. Second, this paper explores the spatial spillover effects and causes of the variability in infrastructure resilience among cities at different time points using a spatial econometric model that considers spatial dependence. Again, this paper takes 283 cities at the prefecture level and above in China as its research objects to more intuitively reflect the spatial heterogeneity of UIR levels at the municipal scale, which is an important socioeconomic management unit.”

Once again, thank you very much for your constructive comments and suggestions, which have helped us a lot to improve the quality of the paper.
